# Exploring the Demographic Factors and Facilitators to Addiction Abstinence

**DOI:** 10.22086/gmj.v0i0.1074

**Published:** 2018-12-10

**Authors:** Maryam Taghva, Ramin Shiraly, Ebrahim Moghimi Sarani

**Affiliations:** ^1^Department of Community Medicine, School of Medicine, Shiraz University of Medical Sciences, Shiraz, Iran; ^2^Department of Community Medicine, School of Medicine, Shiraz University of Medical Sciences, Shiraz, Iran

**Keywords:** Addiction, Abstinence, Substance Abuse, Demographic Factors, Opioid

## Abstract

**Background:**

Accumulating evidence indicates a remarkable increase in substance addiction. Substance abuse and addiction impose severe social, political, economic, cultural and health-related damages on societies. Little is known, however, about demographic factors and facilitators to addiction abstinence. The purpose of the current study was to explore the factors associated with opioid avoidance.

**Materials and Methods:**

This cross-sectional study was performed to record socio-demographic data and facilitating factors to abstinence in 600 interviews, according to data collecting forms, with patients who had drug abuse disorders at Shiraz city during 2016. Correlation test, T-test, and ANOVA were employed for data analysis. P value <0.05 was considered as the significance level.

**Result:**

There was a significant difference between mean abstinence time and demographic factors such as age, sex, occupation and marital status. Also, 53% of people reported that they had the longest abstinence time using the narcotics anonymous (NA) method. TO 33% of patients, the most important facilitating factor in abstinence according to the patient’s opinion was family support.

**Conclusion:**

Individual, social, psychosocial and medical variables affect the abstinence duration of substance abuse. Identifying the factors associated with longer abstinence can be helpful in designing prevention and treatment programs for variables that affect the recurrence.

## Introduction

Addiction and drug abuse are among the most important problems of recent decades that have increased globally. Drug addiction involves a biological process that induces changes in the brain and causes loss of control due to the use of drugs [1]. Addiction is a biopsychosocial disorder; nevertheless, it can be treated like any other disease [2].

According to Sussman *et al*., elements of addiction include engagement in the manner to achieve appetitive effects, temporary enjoyment, loss of control and suffering harmful consequences [3]. The annual report of the World Health Organization (WHO) in 2005 reveals that there are about 255 million people addicted to opiates worldwide and the number of drug abuse victims increases every day [4]. Substance abuse and addiction impose severe social, political, economic, cultural and psychosocial problems, such as an increase in crime, robbery and child abuse [5]. Illicit drug use often associated with other substances that leads to chronic disease, so, addiction becomes an important cause of death [6]. Previous studies show that persons with a drug dependency die on average 22.5 years earlier than those without such dependency [7]. Also, family and social relationships of patients improved after abstinence [2].

Iran suffers from drug abuse due to its neighbors, especially Afghanistan as the largest drug producer in the world [8]. In Iran, substance abuse disorders take the fourth rank in terms of severity and importance after accidents, cardiovascular diseases, and depression [9]. A recent study by the United Nations Office on Drugs and Crime (UNODC) on opiate trafficking shows that Iran reported the largest opiate seizures worldwide in 2014 [10]. There are no direct and standard surveys to estimate the prevalence of drug abuse in Iran, but a nationwide survey carried out in 2007, revealed that there are 1.2 million drug-dependent people in a total population of 75 million [11].

The current study aims to investigate the factors associated with opioid avoidance through interviews with people undergoing treatment as well as those with a history of substances dependency.

## Materials and Methods

### Study Population

This was a cross-sectional study that recorded socio-demographic data and facilitating factors to abstinence. In this study, data were collected by interviewing, according to data collecting forms, for patients who had drug abuse disorders at Shiraz during 2016. The study was approved by the Ethics Committee of Shiraz University of Medical Sciences (approval code: 10657).

### Sample Size Calculation and Participants

Considering the confidence interval (CI), standard deviation (SD) of abstinence duration, decision and design effect equal to 95%, 5.9 (based on the review of articles), 0.56 and 1.5, respectively, the sample size was calculated to be 600 participants. Participants in this study included people with a history of drug dependency that were treated or were under treatment and enrolled with personal satisfaction. Three sources of sampling were used to access these people were addiction treatment clinics (agonist therapy centers), centers for NA, and people with a history of addiction abstinence that had returned to the community and were not under certain treatment (population-based).

In each of the mentioned sources, a method of sampling was appropriate for the number and availability of cases. Because of a large number of patients in addiction treatment clinics (about 8,000 people in 120 centers in Shiraz), cluster sampling method was used. For NA an easy sampling method was applied because of the limited number of these centers in Shiraz. Finally, due to hard access to the people who have classified as group 3 above, snowball sampling method was used.

### Inclusion and Exclusion Criteria

Inclusion criteria were history of substance abuse disorder (based on criteria DSM5 report), any illicit drug (i.e., opioid, heroin, methamphetamine dependence and their derivatives or a collection of these substances), and history of serious attempts to quit drug addiction (medical treatment) or an individual’s serious action for abstinence leading to successful abstinence for at least one month.

Also, subjects who used drugs for fun and did not meet the criteria for substance abuse disorder, addicted to alcohol, and patients who depended only on opioid drugs such as tramadol or methadone were excluded from the study.

### Data Collection

After obtaining the necessary licenses and coordination, data were collected by interviewing patients who had drug abuse disorders, according to data collecting by researcher-designed checklists that were based on factors related to the avoidance of drug abuse. The checklists completed through interviews by a questioner, after face to face training of questioner, for every addicted person who was being treated or person with a history of drug dependence. Also, demographic information includes age, sex, level of education, marital status, occupation, monthly income (the conversion of Iranian Rials to United State dollar was considered), history of type and amount of drug consumption, and history of various methods of treatment that the person had experienced were obtained. Finally, a checklist with an open-ended question was applied to identify the most important facilitating factor in abstinence according to the patient’s opinion (self-report).

The purpose of the study was explained to the patient. Patients were assured that their information would be kept anonymous and secret. The data collection checklists were completed for patients who gave their verbal consent to the interview.

### Data Analysis

After interviews and completion of missing information by the questioner, data were analyzed by SPSS version 16 (IBM SPSS Statistics for Windows, Version 16.0, Released 2007. Armonk, NY: IBM Corp). Descriptive statistics were surveyed to identify respondent’s demographic characteristics. Categorical variables were measured as a percentage while continuous variables were expressed as mean values. A correlation test was used to determine the relationship between the mean abstinence time and the quantitative data age. Also, T-test and ANOVA were applied for qualitative data analysis. Linear regression was used to determine the association between demographic variables and abstinence duration. The stepwise method was applied to determine the significant variables. Because of abstinence duration data did not have a normal distribution, square root transformation was used for normalization. The P <0.05 was considered as the significance level.

## Result

Six hundred interviews were conducted in this study. Two hundred forty-six interviews from addiction treatment centers, 229 interviews from NA groups and 125 interviews were done population-based. The interviewees were aged 15-75 years. The distribution of age data was not normal and had left skewness (median=14; SD=37.3; interquartile rang=30). The age range of 30-45 years had the most frequency. The mean age of participants was 35.47±9.93 years. Some other demographics data show in Table-1.

**Table 1:** Distribution of Demographic Variables and Statistical Measures

**Variables**	**Frequency**	**Percentage**	**P-value**
**Sex**			
Male	518	86.3	<0.001
Female	82	13.7	
**Marital Status**			
Married	351	58.5	<0.001
Single	207	34.5	
Divorced	42	7	
**Occupational status**			
Employed	389	64.8	0.004
Unemployed	211	35.2	
**Monthly income**			
Low (<500 USD)	156	26	0.17
Middle (500-1000 USD)	258	43	
High (>1000 USD)	186	31	
**Educational level**			
High school and diploma	165	27.5	0.09
Associate degree and higher	217	49.5	

**USD:** United States dollar

Also, 36% of the participants were mostly dependent on opium and 26% on heroin, 25.5 on the crystal, and 12.3 on cannabis. The minimum duration of abstinence was one month and the maximum period of abstinence was 360 months. Also, 318 persons (53%) reported the longest abstinence time making use of the NA method while 101 others (16.8%) had the longest time with using methadone maintenance therapy method (Table-2).

**Table 2:** The Frequency of Methods with the Most Abstinence Time Based on Patients Self-Report

**Abstinence Method**	**Frequency**	**Percentage**
**Narcotic anonymous**	318	53
**Methadone maintenance therapy**	101	16.8
**Psychotherapy and psychological counseling**	68	11.3
**Residential treatment in campus**	47	7.8
**Ultra-rapid opioid detoxification**	31	5.2
**Self-therapy**	13	2.2
**Others**	22	3.7

In 200 persons (33.3%) the most important facilitating factor in abstinence according to the patient’s opinion was family support, and in 16% it was personal volition (Table-3). The results of the study showed a weakly direct linear relationship between the patient’s age and the mean duration of abstinence (P<0.001; r=0.33). The mean duration of abstinence was statistically significant by gender and was greater in men (P<0.001; 95%CI: 9.29-12.88). The mean abstinence in both employed and unemployed groups had a statistically significant difference (P=0.004). There was a significant difference between mean abstinence and marital status (P<0.001) such that single people with mean abstinence of 20.82 months had the minimum mean of abstinence compared to married and divorced people. The age coefficient showed that as the age increased, the abstinence duration increased. Also, the gender coefficient showed that men had a longer abstinence duration than women (Table-4).

**Table 3:** The Frequency of the Most Important Facilitating Factors to Addiction Abstinence-Based on Patients Self-Report

**Facilitating factors**	**Frequency**	**Percentage**
**Family support**	245	40
**Self-volition**	114	19
**Addiction drug center advisers**	33	5
**Methadone maintenance therapy**	26	4.3
**Physical illness**	24	4
**Being religious**	12	2
Others*	146	24

* Economic problem, fear of the future, aging, etc.

**Table 4:** Association Between Study Variables and Abstinence Duration

**Variable**	**Regression coefficient (β)**	**Standard error (SE)**	**P-value**
**Age**	0.08	0.01	<0.001
**Sex**	1.25	0.33	<0.001

The stepwise analysis displayed that there was not an association between abstinence duration and other variables.

## Discussion

In this study, some of the demographic variables had a significant difference with the duration of abstinence. The mean age of participants was 35.47 years. In a study in the United States, the prevalence of drug use in the age group of 18-25 had the highest rate such that 78% of drug addicts were under 25 years; about 50% of whom were under 21 years [12]. The causes of higher age of addiction among the participants in this study can be first due to the fact that in the lower age group, a short period of addiction has passed and they have not yet understood the depth of the catastrophe, therefore they are less likely to go to treatment centers to stop their drug addiction. Secondly, fears of legal prosecution may be higher in the younger age, and therefore fewer references occur [13].

The mean duration of abstinence in males and females is statistically significant and is greater in male patients. This result in line with the study by Back *et al*. (2011).

In general, females’ shift from use to dependence is more rapidly than males and also females suffer from more physical and emotional complications of addiction by comparison. Despite this, women refer less frequently to addiction treatment centers. Because little attention is paid to gender differences in addicts, this kind of information can be useful in therapeutic approaches [14]. Three hundred eighteen people (53%) reported the most abstinence time using the NA method.

NA method, the12-Step model, is one of the most well-known and commonly used types of recovery support. The 12-Step method is a way that increases the odds of sustaining abstinence for several years [15]. This method has more benefits than meeting attendance [16].

The result of current study matched the study by Zhou *et al*. (2009) and Jackson *et al*. (2003) who showed that there is not a relationship between education and the success rate of abstinence [17, 18]. It is noteworthy that we typically expect drug abstinence to be better and more successful in a higher educated group and this can be a sign of the defect of current methods in these centers. This defect can be resolved through individual and group training, educational pamphlets, and films. On the other hand, the tendency of educated people to drugs abuse can be a danger to the community, which requires further study [19]. There was a statistically significant difference between the two groups employed and unemployed groups. That means employed people are more likely to have longer abstinence duration. In the study of Seraji *et al*. (2010), unemployment was one of the main causes of drug abuse recurrence. Different researches show the fact that there is a significant correlation between unemployment and addiction, and that, the probability of addiction and recurrence of substance abuse is greater among the unemployed [20]. Unemployment leads to various deviances such as addiction not only through the lack of decent life and the lack of material and spiritual well-being of family members but also through disturbances in personality, moral stability, self-esteem, hope for the future and individual authority [21].

There was not a statistically significant difference between the mean period of abstinence and the type of substance. But, this difference can be considered clinically, meaning that people who consume opium have longer abstinence duration than those who consume other drugs like heroin and crack. Farzam *et al*. study results also showed that the percentage of recurrence in opium users (39.5) is less than heroin users (62.5%) [22]. Perhaps the reason for this can be attributed to the more serious destruction of the defense and support mechanisms of crackers and heroin addicts [23]. On the other hand, addiction to these substances is considered as the final stage of addiction, and such people are usually exiting the cycle of social activity and as a result may be weaker to prevent re-use in the future [24]. It was found that the duration of drug abandonment in a single group varies between divorced and married groups which are consistent with the study of Lowinson *et al*. (1996) who showed that the recurrence of drug use is higher in single populations [23]. This is due to the family’s supportive role in the success of addiction abatement programs. The most critical facilitating factor in abstinence according to the patients’ opinion was family support. This subject is consistent with a meta-analysis by Hoeve *et al*. (2012) that showed a poor relationship with parents caused delinquency in children [25]. The existence of sufficient motivation and commitment to quitting and emotional-psychological support from the family are factors of success in abstinence [26]. The results showed that family support is associated with addiction abstinence. These results recommend that direct family support may help persons to decrease or quit their substance use [27].

It should be noted that sampling from three different sources and multiple centers was one of the strengths of the current study and the unwillingness of some people to participate in the study is one of its limitations.

## Conclusion

The results of the current study show that the individual, social, psychosocial and medical variables are factors that affect the abstinence duration of substance abuse.

Identifying the factors associated with longer abstinence can be helpful in predicting relapse in addicts, and also, in designing prevention and treatment programs for variables that affect the recurrence. It is recommended that similar studies be carried out in other provinces by random sampling to generalize the results at the national level.

## Conflict of Interest

There is not any conflict of interest in this research.
